# Anthropometric Phenotype of Patients with PMM2-CDG

**DOI:** 10.3390/children8100852

**Published:** 2021-09-26

**Authors:** Patryk Lipiński, Agnieszka Różdżyńska-Świątkowska, Anna Bogdańska, Anna Tylki-Szymańska

**Affiliations:** 1 Department of Pediatrics, Nutrition and Metabolic Diseases, The Children’s Memorial Health Institute, 04-730 Warsaw, Poland; a.tylki@ipczd.pl; 2 Anthropology Laboratory, The Children’s Memorial Health Institute, 04-730 Warsaw, Poland; a.rozdzynska-swiatkowska@ipczd.pl; 3 Department of Biochemistry, Radioimmunology and Experimental Medicine, The Children’s Memorial Health Institute, 04-730 Warsaw, Poland; a.bogdanska@ipczd.pl

**Keywords:** congenital disorders of glycosylation, phosphomannomutase 2 deficiency, growth evolution, head circumference, long-term follow-up

## Abstract

Background: Growth failure is commonly reported in children with PMM2-CDG. The aim of the study was to delineate the longitudinal anthropometric phenotype of patients with PMM2-CDG and attempt to find some correlations between the genotype and anthropometric phenotype. Materials and methods: Retrospective chart review of PMM2-CDG patients’ medical records was performed regarding the anthropometric measurements (head circumference, body length/height, body weight, body mass index) and *PMM2* variants. Results: A negative tendency of growth evolution was observed. Patients found to be heterozygous for R141H grew slower than other patients. Body weight was correlated with body height. A negative tendency of the growth rate of head circumference was observed. Patients found to be heterozygous for R141H experienced slower growth than other patients. Conclusions: Long-term observational studies are essential to characterize the anthropometric phenotype. The body growth failure, as well as head circumference growth failure, were more severe in patients found to be heterozygous for R141H.

## 1. Background

Glycosylation is the most common post-translational modification of proteins and lipids. About 50% of human proteins are glycoproteins. They are important components of the hormone cascades, regulating growth and metabolism [1]. 

Phosphomannomutase 2 deficiency (PMM2-CDG) is the most common congenital disorder of glycosylation (CDG) in Poland [2,3,4]. PMM2-CDG is caused by biallelic pathogenic variants in the *PMM2* gene, encoding enzyme phosphomannomutase 2, which catalyzes the second step in the N-glycosylation pathway: the conversion of mannose-6-phosphate to mannose-1-phosphate. PMM2 deficiency results in a multi-organ involvement with a predominant neurological phenotype that could manifest as an isolated neurological syndrome or a neuro-visceral form [5]. Growth failure and failure to thrive are commonly reported in children with PMM2-CDG [5]. However, there is still limited information on the natural history of PMM2-CDG regarding the anthropometric phenotype. Establishing the growth curves for this population is of significant interest, especially to clinicians and families who care for these individuals.

The aim of the study was to delineate the anthropometric phenotype of patients with PMM2-CDG during the long-term follow-up and attempt to find some correlations between the genotype and anthropometric phenotype. 

## 2. Material and Methods

Among the 17 patients diagnosed with PMM2-CDG in our institute, data regarding anthropometric results were available for 9 of them (only for boys due to the paucity of girls’ individual health records). Detailed characteristics on our patients with PMM2-CDG have been previously published [4,6].

Birth body length, weight, and head circumference (OFC) values were obtained from individual health records. Two-tailed *t*-tests were used to compare the mean values between the patients and the healthy population [7]. The data from the 9 boys (number of measurements for height = 36; height SDS from 1.06 to −5.45; number of measurements for head circumference = 23, OFC SDS from 0.04 to −4.13) were divided into 15 calendar age classes. The mean z-scores for each age class were calculated. The degree and direction of deviations of the considered features were analyzed using the data standardization method. The calculated values were presented as z-scores, while the growth trend for body height (available in 7 out of 9 patients; in 2 others, there were available only single growth values) was assessed using the simple linear regression model. The average values of body length/height, OFC, and body mass index (BMI) were calculated to establish the appropriate growth curves.

The molecular analyses results were available for all 9 patients with PMM2-CDG with anthropometric measurements. Based on the genotype, patients from the study group were divided into two groups: group I, including patients found to be a compound heterozygous for the variant c.422G>A,p.(Arg141His) (classified as R141H/other); and group II, including patients with other pathogenic variants (classified as other/other).

## 3. Results

All our patients presented with an infantile neuro-visceral form of PMM2-CDG. The mean age at diagnosis was 4 months, while the mean time of follow up was 10 years, as shown in Table 1. 

### 3.1. Anthropometric Measurements at Birth

All neonates presented with normal OFC. Birth length was available only for boys due to the paucity of girls’ individual health records. Birth length in boys was significantly higher than in the general population. The differences in the mean values for the other parameters were not significant. The mean birth body length, weight, and OFC were calculated and presented in Table 2. 

### 3.2. Anthropometric Assessment during Follow-Up

#### 3.2.1. Growth

**Figure 1:**
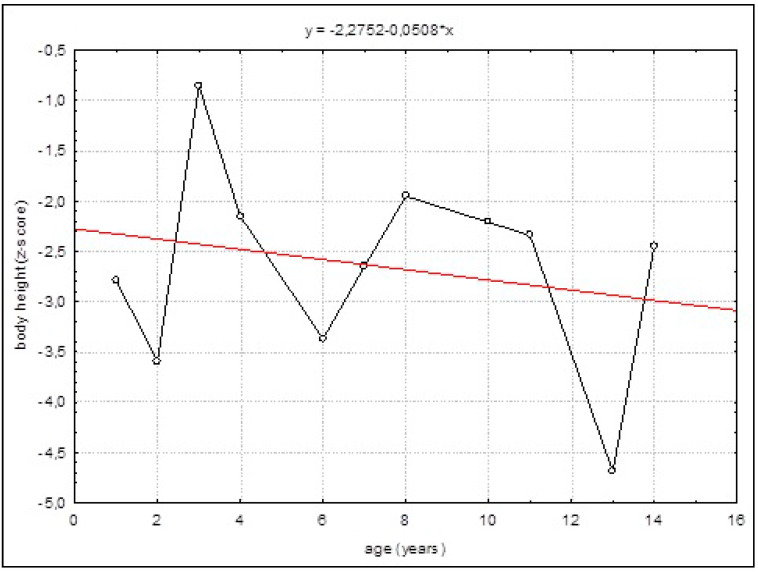
The straight-line regression of standardized body length/height against the adopted reference system.

A negative tendency of growth evolution was observed, as confirmed by the simple linear regression model. This trend was negative but not statistically significant (Figure 1). 

#### 3.2.2. Growth in Relation to Genotype

**Figure 2:**
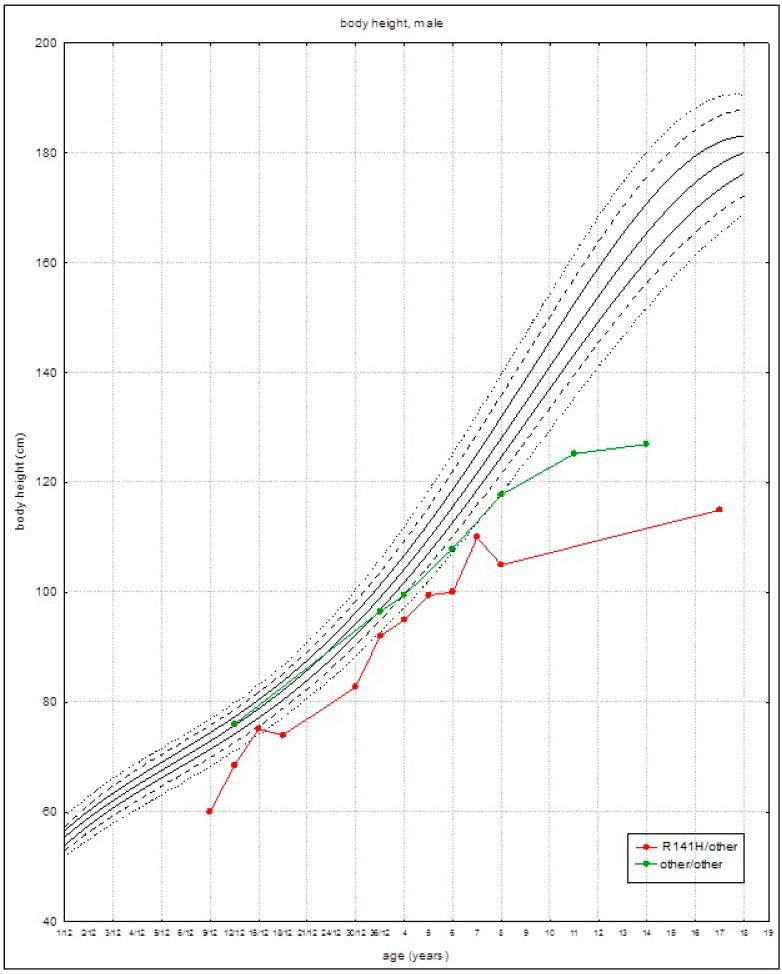
Growth curves (established on the average growth values) for patients with PMM2-CDG for the given genotypes: R141H/other (red marks) and other/other (green marks) on references growth charts for the healthy population.

Patients found to be heterozygous for R141H grew slower than other patients. The growth/height parameters were below the third percentile at about the age of 3 years (Figure 2). Patients from group II (with other than R141H variants) grew slightly faster in the first years of life, and the growth/height parameters were below the third percentile at about 8 years of age (Figure 2). Growth acceleration was not observed during the adolescence in both groups.

#### 3.2.3. Body Weight and BMI

**Figure 3:**
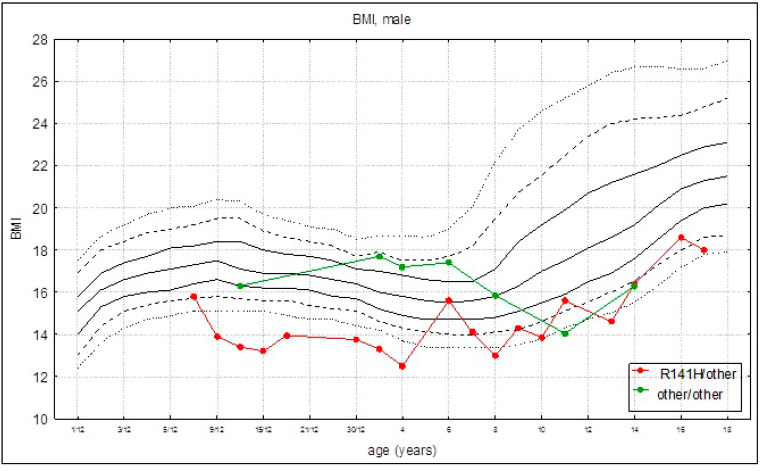
Body mass index (BMI) curves (established on the average BMI values) for patients with PMM2-CDG for the given genotypes: R141H/other (red marks) and other/other (green marks) on references growth charts for the healthy population.

Mean BMI for the whole group was 15.3 (±1.54), while the mean BMI was 14.7 (±1.65) and 16.4 (+0.78) for patients heterozygous for R141H (group I) and patients with other pathogenic variants (group II), respectively. Body weight was in line with body height. In patients found to be heterozygous for R141H, the BMI growth rate was slower than in patients with other *PMM2* variants (Figure 3).

#### 3.2.4. Head Circumference

**Figure 4:**
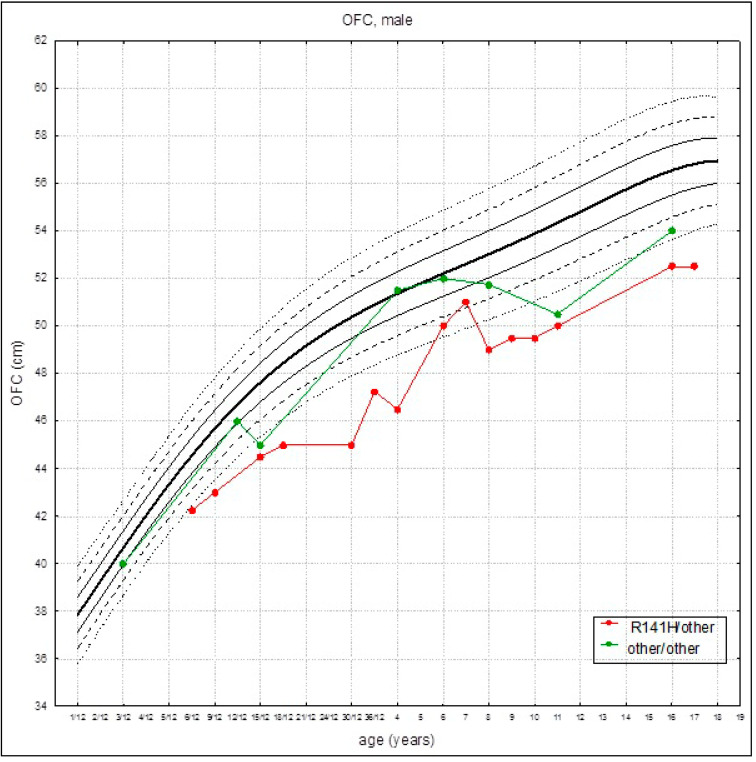
Head circumference curves (established on the average head circumference values) for patients with PMM2-CDG for the given genotypes: R141H/other (red marks) and other/other (green marks) on references growth charts for the healthy population.

**Figure 5:**
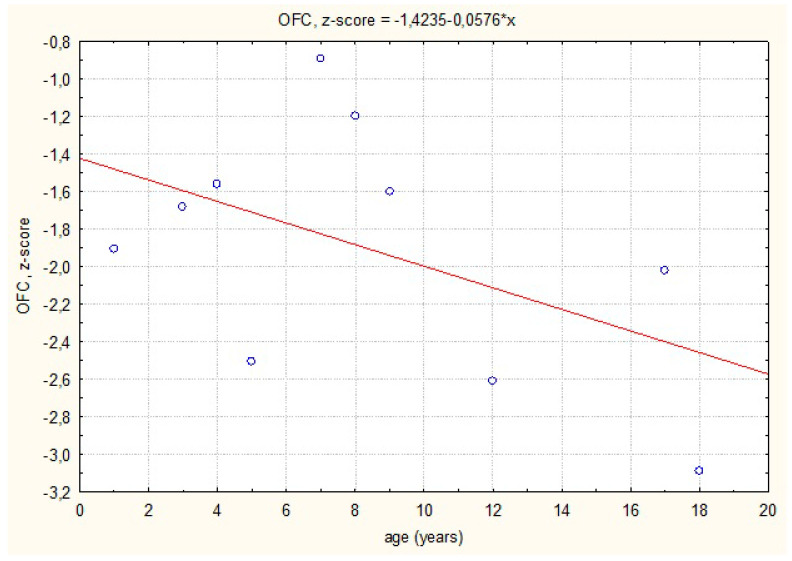
The straight-line regression of standardized head circumference against the adopted reference system.

The growth rate of head circumference was in line with the reference charts in the first months of life. In patients found to be heterozygous for R141H, the growth rate was slower than in patients with other *PMM2* variants (group II), and the OFC were below the third percentile at about 6 months of age (Figure 4). A negative tendency of the growth rate of OFC, confirmed by the simple linear regression model, was observed. This trend was statistically significant (Figure 5).

## 4. Discussion

Although growth failure, as well as failure to thrive and acquired microcephaly, are commonly reported in patients with PMM2-CDG, this study provides a unique analysis of longitudinal data on the anthropometric phenotype and genetic correlations in a group of Polish PMM2-CDG patients. 

In the recently published clinical guidelines on PMM2-CDG, it was estimated that about half of reported patients presented with a short stature [5]. After a normal anthropometry at birth, a postnatal growth decline was commonly observed. Surprisingly, the birth length of our patients (boys) was significantly higher than in the general Polish population. However, a negative growth trend for body height was observed, similarly to the literature, and all our patients presented with a short statute.

So far, there have been three studies published regarding the longitudinal anthropometric assessment of PMM2-CDG patients.

Kjaergaard et al. provided an analysis of a prepubertal growth in 25 PMM2-CDG patients (mean age: 7.6 years) of Danish origin with a homogeneous (R141H/F119L) genotype [8]. Measurements of length, weight, and head circumference were within the normal ranges at birth, and the mean values of weight and length declined during the first 7 months of life. Mean length/height and weight stabilized or increased slightly during the late infancy and childhood but did not return to the normal ranges by the age of 10 years. 

Monin et al. described a cohort of 29 French patients aged from 15 to 49 years (mean age: 27.4 years). The researchers observed a clear delay in growth, weight, and head circumference [9]. During the follow-up, there was no significant improvement in these parameters. 

In the study of Witters et al. conducted on 75 PMM2-CDG patients (mean age: 11.02 ± 6.91 years), a clear delay in growth, weight, and head circumference was also observed [10]. Besides two patients, all had compound heterozygous pathogenic variants, with R419H most frequently observed. During the follow-up (7.4 ± 4.5 years), a deterioration in growth was observed. This was in contrast to IGF, which showed an age-related increase.

Our observations are similar to the abovementioned results. However, this is the first report on the body growth, head circumference growth, and BMI growth rates in relation to the PMM2-CDG genotype. The pathogenic variant c.422G>A,p.(Arg141His) is the most frequently identified in patients with PMM2-CDG. Homozygosity for this pathogenic variant has been shown to be absent since it is probably lethal. In the present study, we observed that patients found to be heterozygous for c.422G>A,p.(Arg141His), showed lower body growth, head circumference growth, and BMI growth rates. It is worth noting that, due to the small study group, the conclusions are difficult to generalize. Thus, multicenter studies to collect more data will be essential to draw more definitive conclusions.

The exact mechanism of the growth failure in PMM2-CDG is unknown. It is suggested that the impairment of the GH-IGF-1 cascade may play an important role. The previous in vitro and in vivo studies have shown the importance of glycosylation in the stability and function of the components of the IGF ternary complex [11]. PMM2-CDG patients have lower serum levels of IGF-1, IGFBP3, and acid-labile subunit despite normal or increased levels of growth hormones [5]. Miller et al. (2013) reported a child with PMM2-CDG with clinical improvement of growth following rhIGF-1 therapy [12]. Unfortunately, we have no data on IGF1 or IGFBP3 to support the hypothesis about the supposedly more severely affected carriers of the R141H allele.

## 5. Conclusions

This is a manuscript on the growth evolution in a small number of PMM2-CDG patients. A novel feature is the finding that patients heterozygous for the R141H showed a slower growth than patients with other genotypes. Long-term observational studies are essential to characterize the anthropometric phenotype.

## Figures and Tables

**Table 1:** Characteristics of the study group.

Patient’s Number/Sex	Genotype	Classification of the Study Group	Age at Diagnosis	Duration of Follow-Up
1/M	c.169G>A,p.Gly57Arg/ c.422G>A,p.Arg141His	R141H/other	5 m	4 y 4 m
2/M	c.691G>A,p.Val231Met/ c.640-15479C>T (deep intronic splice site mutation)	Other/other	2 m	14 y 2 m
3/M	c.338C>T,p.Pro113Leu/ c.470T>C,p.Phe157Ser	Other/other	1 m	18 y
4/M	c.24delC,p.C9AfsX27/ c.385G>A,p.Val129Met	Other/other	1 m	3 y 6 m
5/M	c.357C>A, p.Phe119Leu/ c.422G>A p.Arg141His	R141H/other	6 m	6 y 8 m
6/M	c.422G>A,p.Arg141His/ c.691G>A,p.Val231Met	R141H/other	3 m	13 y 6 m
7/M	c.422G>A,p.Arg141His/ c.484C>T,p.Arg162Trp	R141H/other	6 m	17 y 3 m
8/M	c.155T>G,p.Val52Gly/ c.640-23A>G,p.?	Other/other	6 m	8 y
9/M	c.422G>A,p.Arg141His/ c.691G>A,p.Val231Met	R141H/other	2 m	2 y 4 m

Abbreviations: y—years; m—months.

**Table 2:** Mean birth parameters in comparison with the male general population. Body length: 52.2 cm; body weight: 3.5 kg; OFC: 35.3 cm.

Body Length (cm)	*p* Value	Body Weight (kg)	*p* Value	OFC (cm)	*p* Value
56.1 ± 2.03	0.01	3.47 ± 0.51	0.57	34.9 ± 1.28	0.84

## Data Availability

All data generated or analysed during this study are included in this published article.
